# Septicemia Caused by Tick-borne Bacterial Pathogen *Candidatus* Neoehrlichia mikurensis

**DOI:** 10.3201/eid1607.091907

**Published:** 2010-07

**Authors:** Jan S. Fehr, Guido V. Bloemberg, Claudia Ritter, Michael Hombach, Thomas F. Lüscher, Rainer Weber, Peter M. Keller

**Affiliations:** Author affiliations: University Hospital, Zurich, Switzerland (J.S. Fehr, T.F. Lüscher, R. Weber);; University of Zurich, Zurich (G.V. Bloemberg, C. Ritter, M. Hornbach, P.M. Keller)

**Keywords:** Candidatus Neoehrlichia mikurensis, septicemia, human infection, 16S rRNA gene PCR, therapy, tick-borne pathogen, bacteria, dispatch

## Abstract

We have repeatedly detected *Candidatus* Neoehrlichia mikurensis, a bacterium first described in *Rattus norvegicus* rats and *Ixodes ovatus* ticks in Japan in 2004 in the blood of a 61-year-old man with signs of septicemia by 16S rRNA and *groEL* gene PCR. After 6 weeks of therapy with doxycycline and rifampin, the patient recovered.

Since the novel bacterial genus *Neoehrlichia* wass first described in 2004, its pathogenic role in humans has remained unexplained (*1*). Related bacteria such as *Ehrlichia chaffeensis* and *Anaplasma phagocytophilum* are emerging tick-borne human pathogens that cause monocytic and granulocytic ehrlichiosis, respectively. These tick-borne diseases manifest themselves as febrile illness, mild transient hepatitis, transient thrombocytopenia, and occasionally as a rash. The family *Anaplasmataceae* compromises the genera *Ehrlichia*, *Anaplasma*, *Neorickettsia*, and *Aegyptianella*, and the proposed genus *Neoehrlichia.* These are all obligate intracellular bacteria, which currently are difficult or impossible to isolate and culture (*2*). Infections caused by agents of this bacterial family have been recognized as an emerging problem in the past 2 decades, possibly due to ecologic changes and the resulting expansion of tick populations (*3*).

## Case Report

In August 2009, a 61-year-old Caucasian man who lived in Switzerland sought treatment at the emergency department of University Hospital in Zurich, reporting a 10-day history of malaise, temperature as high as 39.5°C, chills, and moderate dyspnea. Six weeks previously, he had undergone coronary artery bypass graft surgery and mitral valve reconstruction for which prosthetic material was used. The patient had not noticed tick bites or a skin rash; neither did he recall a rodent bite. A pet dog and cat lived in his household.

Physical examination showed a reduced general health condition and a temperature of 38.5°C. Blood pressure was 109/68 mm Hg, heart rate was 86 beats/min, and oxygen saturation was 95% with 2 L nasal oxygen. No murmur was detected on cardiac auscultation. No skin or joint abnormalities were found. Laboratory tests showed elevated leukocytes (12.9 × 10^3^ cells/µL), with a high fraction of neutrophils (10.1 × 10^3^ cells/µL) and thrombocyte count within reference range (277 × 10^3^ cells/µL); aminotransferase levels within reference ranges (aspartate aminotransferase 18 U/L, alanine aminotransferase 20 U/L); and an elevated C-reactive protein (CRP) of 68 mg/L (reference range <5 mg/L). Chest radiograph showed no signs of cardiac decompensation or of pulmonary infiltrates. Transthoracic echocardiograph showed only minor insufficiency of the aortic and tricuspid valves. In addition, degenerative alterations of aortic valve, but no vegetations, were noted with comparable findings in the follow-up echocardiograph 1 week later.

At the follow-up visit, no hints of infectious foci were found. Five sets of blood cultures were drawn with >12 h difference between the first and the last set. Antimicrobial drug treatment for endocarditis with prosthetic material, consisting of vancomycin, gentamicin, and rifampin, was initiated.

Blood cultures remained negative for microbial growth, even after extended incubation. Serologic tests for agents of culture-negative endocarditis and tick-borne diseases were performed. Enzyme immunoassays (EIAs) were positive for immunoglobulin (Ig) G antibodies reactive to *Bartonella henselae* (512) and *B. quintana* (1,024), *Coxiella burnetii* (phase II IgG titer 160), *Rickettsia rickettsii/conorii* (IgG 256), and *Rickettsia typhi* (IgG 128), *Mycoplasma pneumoniae* (index 2.7). IgM was positive only for *A.* phagocytophilum (512, atypical fluorescence pattern), presenting a low titer of IgG at this stage. Serologic test results for *Brucella* spp.*, Chlamydia trachomatis, Chlamydia pneumoniae,* and *Borrelia burgdorferi* were negative. Species-specific PCRs for *A.* phagocytophilum, *Tropheryma whipplei*, *B. henselae*, *B. quintana*, *Legionella* spp., and *L. pneumophila* were negative.

**Figure 1:**
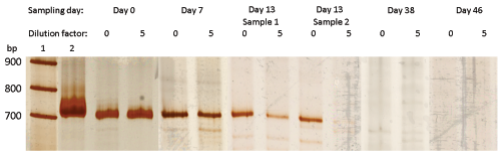
Polyacrylamide gel electrophoresis (SDS-PAGE) analysis of broad range 16S rRNA gene PCR products obtained from blood samples. Lane 1, marker, 100 bp DNA ladder (Roche DNA Marker XIV); lane 2, positive control, *Escherichia coli*; following lanes, PCR products obtained from blood specimens arranged by date of collection. For each specimen PCR products are shown obtained with undiluted (0) and 5×-diluted (5) DNA extracts. The 2 last negative samples are not shown.

**Figure 2:**
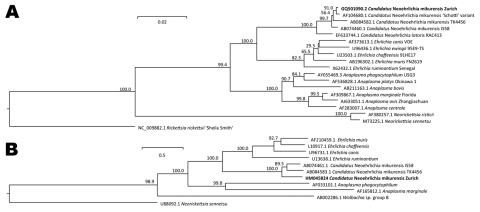
A) Phylogenetic tree based on the 16S rRNA gene sequences of *Candidatus* Neoehrlichia mikurensis GQ501090.2 (our patient’s isolate, herein termed Zurich and indicated in **boldface**) and related organisms. The number at nodes indicates percentages of bootstrap support based on 10,000 replicates. Scale bar indicates 0.02 substitutions per nucleotide position. B) Phylogenetic tree based on the *groEL* sequences. Scale bars indicate 0.05 substitutions per nucleotide position.

Bacterial broad-spectrum 16S rRNA gene PCR, followed by sequence analysis, identified *Candidatus* Neoehrlichia mikurensis in 4 of 8 sequential blood samples; the 4 samples that tested positive were collected before (day 0) and during the initial phase (days 7 and 13) of an effective course of antimicrobial drug therapy (Figure 1). For 16S rRNA gene amplification, DNA was extracted and amplified from anticoagulated blood (4-mL EDTA tubes), uncoagulated blood from a BacT/ALERT SA aerobic blood culture flask (bioMérieux SA, Geneva, Switzerland), and coagulated blood (Technical Appendix, www.cdc.gov/eid/content/16/7/1127-Techapp.pdf) as described (*4*). Sequences derived (GenBank accession nos. GQ501089-GQ501091) were analyzed by SmartGene IDNS software (Zug, Switzerland). We amplified and sequenced (1,150 bp; GenBank accession no. HM045824) an alternative target gene (*groEL*) with primers (Technical Appendix) derived from published sequences (*1*), which showed 98% homology to previously published *Candidatus* Neoehrlichia mikurensis *groEL* sequences (Figure 2, panel B).

After being treated with vancomycin, gentamicin, and rifampin for 10 days, the patient became afebrile, and clinical symptoms improved. Leukocyte count was within reference range, and CRP dropped from 68 mg/L to 23 mg/L. At this time, *Candidatus* Neoehrlichia mikurensis was detected in the first blood sample. Rifampin (450 mg 2×/d) was continued, and vancomycin and gentamicin were switched to oral doxycycline (100 mg 2×/d). Three weeks later, CRP was 1 mg/L, body temperature was within the normal range, and treatment was continued to finish a 6-week course. Two weeks after the end of treatment, the patient was seen for a follow-up visit. Neither clinical nor laboratory results raised any concern of relapse. Results of broad-range PCR of the 16S rRNA gene to detect *Candidatus* Neoehrlichia mikurensis were negative for the first time in 5 weeks since treatment began initiation and remained negative at the follow-up visit 2 weeks after the end of treatment.

*Candidatus* Neoehrlichia mikurensis was previously found in *Rattus norvegicus* rats and *Ixodes ovatus* ticks in Japan (*1*), in *R. norvegicus* rats in China (*5*), and in *I. ricinus* ticks in the Netherlands (*6*,*7*), Slovakia (*8*), and the Asian part of Russia (*9*). Closely related rickettsial bacteria (Figure 2) have been identified in *Procyon lotor* raccoons in the Piedmont region of Georgia, USA (*10*). Another closely related species (*Candidatus* Ehrlichia walkeri) has been detected in *I. ricinus* ticks collected from humans in northern Italy (*11*). The geographic distribution of the tick population has also been studied (*12*).

Our patient lives in a high-risk area for ticks in Switzerland. *I. ricinus* is the main tick species in this region. A tick-borne disease appears epidemiologically possible in this patient, who is a golfer and the owner of a large garden and thus is repeatedly exposed to the habitat of the potential vector, *I. ricinus*, even though he remembered no tick bites. Of note, only 50%–70% of patients with Lyme disease remember receiving a tick bite (*13*). Blood of the patient’s pet animals (dog and cat) was examined by broad-range 16S rRNA gene PCR to exclude presence of bacterial pathogens.

In Wister rats, *Candidatus* Neoehrlichia mikurensis has been shown to infect spleen sinus endothelial cells, forming intracellular inclusions on the side of the endosome (by electron microscopy 60 days after infection) (*1*). Accordingly, we assumed that in human hosts, valvular endothelial cells are likely involved. The initial antimicrobial drug therapy, which contained rifampin, may already have contributed to the reduction of the bacterial load but was not completely effective (Figure 1). Thus, following the recommended guidelines for treatment of intracellular rickettsial bacteria with endocardial involvement, we changed to a 6-week course of treatment consisting of rifampin combined with doxycycline (*14*). After the end of the course, we observed a successful response.

We detected *Candidatus* Neoehrlichia mikurensis in 4 of 8 consecutive blood specimens, and repeated analysis showed the disappearance of the pathogen’s DNA during the course of treatment (Figure 1). Laboratory diagnosis of ehrlichiosis is severely hampered because the relevant pathogens cannot be cultured on routine media. Serologic tests depends on samples collected during acute phase of illness, obtaining comparative samples in the course of the disease, and demonstrating a >4-fold increase in antibody titers.

## Conclusions

We have identified *Candidatus* Neoehrlichia mikurensis in multiple blood samples of a patient who sought treatment for septicemia. Therapeutic success has been shown over time by the fact that the suggested pathogen’s DNA was no longer detectable and by a favorable clinical outcome. Surveys of arthropod populations should be conducted to examine the geographic distribution of *Candidatus* Neoehrlichia mikurensis, and species-specific assays could determine the relevance of this organism in human ehrlichial diseases.

## Supplementary Material

Technical Appendix Supplementary Methods: Bacterial DNA Isolation, Bacterial Broad-Range 16S rDNA and groEL PCR.
