# Supplementary material for: Septicemia Caused by Tick-borne Bacterial Pathogen Candidatus Neoehrlichia mikurensis
Source: Emerg Infect Dis. 2010 Jul;16(7):1127–9. doi: 10.3201/eid1607.091907 (PMC3358111; doi:10.3201/eid1607.091907)
Supplement: Technical Appendix — Supplementary Methods: Bacterial DNA Isolation, Bacterial Broad-Range 16S rDNA and groEL PCR. [file 09-1907-Techapp.pdf]

DOI: 10.3201/eid1607.091907

Septicemia Caused by Tick-borne Bacterial Pathogen *Candidatus* Neoehrlichia mikurensis

Technical Appendix

Supplementary Methods

Bacterial DNA Isolation

For the EDTA-blood mixture, the following procedure was applied: 1.5 mL of anticoagulated blood was mixed with 5 mL of EC lysis buffer (155 mmol/L NH_4Cl, 10 mmol/L $KHCO_3$, 2 mmol/L EDTA) in a 50-mL centrifugation tube and cooled on ice for 30 min. The tubes were inverted every 5 min and centrifuged at 16,000 × *g* for 15 min at 4°C. Thel supernatant was discarded, 5 mL EC lysis buffer was added, and the tube was centrifuged at 16,000 × *g* for 15 min. All but 1 mL of the supernatant was discarded; the pellet was gently resuspended and transferred into a 1.5-mL screw-cap tube (Eppendorf, Hamburg, Germany). The tube was centrifuged at 16,000 × *g* for 10 min. Subsequently, the supernatant was removed, and the pellet was mixed with 200 µL of digest buffer (50 mmol/L Tris-HCl pH 8.0, 1 mmol/L EDTA, 0.5% sodium dodecyl sulfate, 400 µg/mL proteinase K [recombinant, PCR-grade; Roche Diagnostics, Rotkreuz, Switzerland]). The suspension was vortexed until the pellet had completely dissolved and then incubated in a thermomixer at 55°C at 1,000 rpm for 2 h.

DNA from a uncoagulated (heparinized) blood sample was prepared as follows: buffy-coat blood was frozen and thawed; 1.5 mL of fluid was then transferred to a 50-mL centrifugation tube, together with 5 mL of EC lysis buffer. The sample was kept on ice for 30 min, and afterwards the tube was centrifuged at 16,000 × *g* for 10 min. Further steps were performed as mentioned for EDTA-blood samples.

To extract DNA from blood culture material, the following protocol was used: 500 µL of blood culture material was added to 1 mL of 50 mmol/L NaOH, 50 mmol/L sodium citrate in a 1.5-mL Eppendorf reaction tube and inverted gently for 10 min. After centrifugation at 16,000 ×

g for 5 min, the supernatant was disposed and the pellet was resuspended in 500 µL 0.5 mol/L Tris-HCl (pH 8.0). Centrifugation was repeated, the supernatant was discarded, and again 500 µL Tris-HCl solution was added and centrifugation was repeated. The supernatant was discarded, and the pellet was resuspended in 200 µL of Tris-EDTA buffer. The solution was incubated in a thermomixer at 95°C at 600 rpm for 1 h. The tube was then centrifuged at 9,300 × *g* for 15 min, and the DNA-containing supernatant was used for further purification steps. The DNA of all samples was finally purified and enriched by using the QIAamp DNA Blood Kit (QIAGEN, Hombrechtikon, Switzerland) with a slightly modified protocol: 200 µL buffer AL was added, the mixture was heated to 70°C for 10 min. Then 200 µL ethanol was added, and the solution was pipetted onto the purification column. The column was centrifuged at 6,000 × *g* for 1 min. The column was transferred to a new collection tube and washed by using 500 µL of buffer AW1, centrifuged for 1 min at 6,000 × *g*, washed with 500 µL of AW2, and centrifuged for 3 min at 16,000 × *g*. Finally, DNA was eluted by using 50 µL of buffer AE. The column was incubated for 5 min and then centrifuged at 6,000 × *g* for 1 min.

Bacterial Broad-Range 16S rDNA and *groEL* PCR

Five microliters of the resulting DNA-containing eluate and 5 µL of a 1:5 diluted eluate were used as template in a 50-µL amplification reaction essentially as described (*1*). Five microliters of *Escherichia coli* chromosomal DNA (≈50 ng/µL) served as a positive control. Briefly, the PCR used 1.25 U low DNA AmpliTaq DNA polymerase (Applied Biosystems, Rotkreuz, Switzerland) in a reaction mixture containing 1× PCR buffer (10 mmol/L Tris-HCl pH 9.0, 50 mmol/L KCl, 1.5 mmol/L $MgCl_2$, 0.1% Triton X), 0.2 mmol/L dNTPs (Roche Diagnostics), and primers at a concentration of 0.1 mmol/L (BAK11w, 5′-AGT TTG ATC MTG GCT CAG-3′; and BAK2 5′-GGA CTA CHA GGG TAT CTA AT-3′). All reagents (e.g., *Limulus* amebocyte lystate water) and buffers used were included as negative controls. The primary amplicons were visualized by polyacrylamide gel electrophoresis and silver staining and reamplified in a seminested PCR with primers BAK11w and BAK553r (5′-TTA CCG CGG CTG CTG GCA C-3′). Automated cycle sequencing was performed by using primer BAK11w on an ABI Prism 3100-Avant Genetic Analyzer (Applied Biosystems). *groEL* was amplified by using primers groEL_F 5′-TAT AGC TAA GGA AGC ATA GTC TA-3′ and groEL_R 5′-AGC TCT

AGT AGC ATG TAA AGC-3′ (this study). Sequencing was conducted with the amplification primers and additional internal *groEL* primers (*1*).

Reference

1. Takano A, Ando S, Kishimoto T, Fujita H, Kadosaka T, Nitta Y, et al. Presence of a novel *Ehrlichia* sp. in *Ixodes granulatus* found in Okinawa, Japan. Microbiol Immunol. 2009;53:101–6. Medline DOI: 10.1111/j.1348-0421.2008.00093.x
